# Supplementary material for: Targeting miR‐34a/Pdgfra interactions partially corrects alveologenesis in experimental bronchopulmonary dysplasia
Source: EMBO Mol Med. 2019 Feb 15;11(3):e9448. doi: 10.15252/emmm.201809448 (PMC6404112; doi:10.15252/emmm.201809448)
Supplement: Supplementary file 1 — Appendix [file EMMM-11-e9448-s001.pdf]

APPENDIX

Manuscript title:	Targeting miR-34a/*Pdgfra* interaction partially corrects alveologenesis in experimental bronchopulmonary dysplasia
First author:	Jordi Ruiz-Camp
**Supplementary Item Number**	**Title or Caption**
Appendix Table S1	Stereological analysis of lungs from P14 miR-34a$^{-/-}$ mice exposed either to normoxia or hyperoxia compared to wild-type controls.
Appendix Table S2	Stereological analysis of lungs from P14 miR-34bc$^{-/-}$ mice exposed either to normoxia or hyperoxia compared to wild-type controls.
Appendix Table S3	Stereological analysis of lungs from P14 tamoxifen-treated *Pdgfra*-CreER^{T2}/miR-34a$^{fl/fl}$ (to generate *Pdgfra*-CreER^{T2}/miR-34a$^{i\Delta PC/i\Delta PC}$) mice exposed either to normoxia or hyperoxia compared to tamoxifen-treated *Pdgfra*-CreER^{T2}/miR-34a$^{wt/wt}$ controls.
Appendix Table S4	Stereological analysis of lungs from P14 wild-type mice treated with a target site blocker cocktail targeting the miR-34a/*Pdgfra* interaction exposed to hyperoxia, compared to scrambled target site blocker-treated controls
Appendix Table S5	Stereological analysis of lungs from P14 wild-type mice treated with antimiR-34a exposed either to normoxia or hyperoxia, compared to scrambled antimiR-treated controls.
Appendix Table S6	Summary of the gene expression microarray data examining changes in mRNA levels after antimiR-34a or scrambled antimiR treatment of mice with concomitant normoxia or hyperoxia exposure.
Appendix Table S7	Real-time RT-PCR primers employed in this study.
Appendix Table S8	PCR primers employed for genotyping in this study.
Appendix Table S9	Antibodies employed in this study.
Appendix Figure S1	Loss of one member of the miR-34 family does not lead to compensatory upregulation of expression of other miR-34 family members.
Appendix Figure S2	Expression of miR-34a was detected in the developing septa and airway epithelium in mouse pups.
Appendix Figure S3	Exposure of the MLg mouse lung fibroblast cell line to 85% O_2 results in pronounced downregulation of expression of *Pdgfra* and *Acta2*.
Appendix Figure S4	Exposure of mice to 85% O_2 downregulates *Pdgfra* and *Acta2* expression in PDGFRα^+ cells isolated from mouse lungs by flow cytometry.
Appendix Figure S5	Repetition of the experiment presented in Fig. 2F.
Appendix Figure S6	Validation of a target site blocker cocktail.
Appendix Figure S7	Gating strategy for the quantification of PDGFRα+ and PDGFRα^+/αSMA$^+$ cells by flow cytometry in mouse lungs after target site blocker application.
Appendix Figure S8	Administration of an antimiR directed against miR-34a predominantly impacts miR-34a expression in mice on day five of life.
Appendix Figure S9	The abundance of α smooth muscle actin (SMA)-positive cells and total abundance of αSMA in mouse lung is not impacted by exposure of newborn mice to hyperoxia.
Appendix Figure S10	Gating strategy for the quantification of PDGFRα^+ and PDGFRα^+/αSMA$^+$ cells by flow cytometry in mouse lungs after antimiR-34a application.
Appendix Figure S11	Administration of an antimiR directed against miR-34a impacts elastin deposition and remodeling during exposure of newborn mice to hyperoxia.
Appendix Figure S12	Determination of the apoptosis status of PDGFRα^+ cells in developing mouse lungs.
Appendix Figure S13	Attempt at the determination of the proliferation of status of PDGFRα^+ cells in developing mouse lungs by S-phase analysis.
Appendix Figure S14	Determination of the impact of hyperoxia and miR-34a on apoptosis and proliferation of primary mouse lung fibroblasts.
Appendix Figure S15	Neither hyperoxia exposure nor antimiR-34a treatment impacts total number of apoptotic status of alveolar type I cells.
Appendix Figure S16	Administration of an antimiR directed against miR-34a does not have a detectable impact on mRNA expression in developing mouse lungs exposed to hyperoxia.

Appendix Table S1. Stereological analysis of lungs from P14 miR-34a$^{-/-}$ mice exposed either to normoxia or hyperoxia compared to wild-type controls.

**Parameter**	**21% O_2**		**85% O_2**			
	**WT**	**miR-34a$^{-/-}$**	**WT**		**miR-34a$^{-/-}$**	
	mean ± SD	mean ± SD	mean ± SD	*P* value *vs.* WT/21% O_2	mean ± SD	*P* value *vs.* WT/85% O_2
V (lung) [cm^3]	0.24 ± 0.02	0.23 ± 0.02	0.21 ± 0.02	0.119	0.23 ± 0.02	0.301
CV [*V* (lung)]	0.074	0.083	0.089		0.102	
V_V (par/lung) [%]	94.19 ± 1.44	91.08 ± 5.60	91.86 ± 1.14	0.715	92.10 ± 3.65	0.999
V_V (non-par/lung) [%]	5.81 ± 1.44	8.92 ± 5.60	8.14 ± 1.14	0.715	7.90 ± 3.65	0.999
N (alv, lung) 10^6	4.52 ± 0.48	4.23 ± 0.33	1.35 ± 0.15	< 0.0001	2.03 ± 0.17	0.021
N_V (alv/par) 10^7 [cm^{-3}]	1.98 ± 0.14	2.00 ± 0.17	0.70 ± 0.12	< 0.0001	0.94 ± 0.06	0.047
CV [N (alv/lung)]	0.10	0.08	0.11		0.12	
S_V [cm^{-1}]	872.60 ± 19.17	872.10 ± 31.63	502.70 ± 60.11	< 0.0001	522.30 ± 26.75	0.844
S (alv epi, lung) [cm^2]	199.30 ± 16.20	180.60 ± 10.79	97.49 ± 12.50	< 0.0001	112.50 ± 13.60	0.323
CV [S (alv epi, lung)]	0.08	0.06	0.13		0.12	
V_V (alv air) [%]	47.78 ± 5.32	49.34 ± 10.11	70.55 ± 4.16	0.0002	77.08 ± 2.76	0.378
V (alv air, lung) [cm^3]	0.11 ± 0.02	0.10 ± 0.02	0.14 ± 0.02	0.055	0.17 ± 0.01	0.051
CV [*V* (alv air, lung)]	0.15	0.16	0.12		0.08	
V (sep, lung) [cm^3]	0.12 ± 0.01	0.11 ± 0.03	0.06 ± 0.01	0.0001	0.05 ± 0.01	0.891
CV [*V* (sep, lung)]	0.12	0.25	0.14		0.20	
τ (sep) [µm]	10.73 ± 0.51	10.21 ± 0.62	12.17 ± 0.23	0.0024	8.69 ± 1.14	< 0.0001
CV [τ (sep)]	0.05	0.06	0.02		0.13	
MLI [µm]	21.91 ± 2.57	22.59 ± 4.34	57.05 ± 9.67	< 0.0001	59.13 ± 3.01	0.936
CV [MLI]	0.08	0.14	0.16		0.05	

Abbreviations: *alv*, alveoli; *alv air*, alveolar airspaces; *alv epi*, alveolar epithelium; *CV*, coefficient of variation; MLI, mean linear intercept; *N*, number; N_V, numerical density; *non-par*, non-parenchyma; *par*, parenchyma; *S*, surface area; S_V, surface density; τ (sep), arithmetic mean septal thickness; *V*, volume; V_V, volume density; WT, wild-type. Values are presented as mean ± SD, $n = 5$ lungs for each group. A one-way ANOVA with Tukey's *post-hoc* analysis was used to determine *P* values.

Appendix Table S2. Stereological analysis of lungs from P14 miR-34bc$^{-/-}$ mice exposed either to normoxia or hyperoxia compared to wild-type controls.

**Parameter**	**21% O_2**		**85% O_2**			
	**WT**	**miR-34b/c$^{-/-}$**	**WT**		**miR-34b/c$^{-/-}$**	
	mean ± SD	mean ± SD	mean ± SD	*P* value *vs.* WT/21% O_2	mean ± SD	*P* value *vs.* WT/85% O_2
V (lung) [cm^3]	0.24 ± 0.01	0.24 ± 0.02	0.21 ± 0.01	0.991	0.21 ± 0.01	0.995
CV [*V* (lung)]	0.07	0.09	0.07		0.03	
V_V (par/lung) [%]	92.73 ± 1.45	93.93 ± 6.46	89.50 ± 1.86	> 0.996	87.96 ± 6.73	0.964
V_V (non-par/lung) [%]	7.27 ± 1.45	7.07 ± 6.46	10.50 ± 1.86	> 0.996	1.20 ± 6.73	0.964
N (alv, lung) 10^6	4.34 ± 0.48	4.24 ± 0.13	1.33 ± 0.13	0.948	1.37 ± 0.09	0.963
N_V (alv/par) 10^7 [cm^{-3}]	1.97 ± 0.26	1.95 ± 0.14	0.71 ± 0.14	0.992	0.74 ± 0.55	0.988
CV [N (alv/lung)]	0.11	0.03	0.10		0.07	
S_V [cm^{-1}]	896.44 ± 51.35	924.42 ± 94.76	559.06 ± 64.74	0.893	566.77 ± 21.3	0.994
S (alv epi, lung) [cm^2]	197.35 ± 8.71	200.53 ± 5.21	106.33 ± 8.21	0.949	105.26 ± 9.94	0.996
CV [S (alv epi, lung)]	0.04	0.02	0.07		0.10	
V_V (alv air) [%]	49.98 ± 5.12	50.71 ± 4.93	67.04 ± 6.62	0.992	69.49 ± 3.27	0.955
V (alv air, lung) [cm^3]	0.11 ± 0.01	0.11 ± 0.01	0.12 ± 0.02	> 0.995	0.13 ± 0.01	0.991
CV [*V* (alv air, lung)]	0.12	0.15	0.14		0.13	
V (sep, lung) [cm^3]	0.11 ± 0.01	0.10 ± 0.01	0.06 ± 0.01	0.981	0.05 ± 0.01	0.917
CV [*V* (sep, lung)]	0.11	0.10	0.21		0.27	
τ (sep) [µm]	11.15 ± 0.86	10.73 ± 1.31	11.75 ± 1.49	0.992	10.78 ± 0.91	0.908
CV [τ (sep)]	0.07	0.12	0.19		0.29	
MLI [µm]	22.42 ± 3.31	22.18 ± 3.73	48.08 ± 11.81	0.998	49.10 ± 4.03	0.997
CV [MLI]	0.14	0.16	0.13		0.13	

Abbreviations: *alv*, alveoli; *alv air*, alveolar airspaces; *alv epi*, alveolar epithelium; *CV*, coefficient of variation; MLI, mean linear intercept; *N*, number; N_V, numerical density; *non-par*, non-parenchyma; *par*, parenchyma; *S*, surface area; S_V, surface density; τ (sep), arithmetic mean septal thickness; *V*, volume; V_V, volume density; WT, wild-type. Values are presented as mean ± SD, *n* = 5 lungs for each group. A one-way ANOVA with Tukey's *post-hoc* analysis was used to determine *P* values.

Appendix Table S3. Stereological analysis of lungs from P14 tamoxifen-treated *Pdgfra*-CreER^{T2}/miR-34a$^{fl/fl}$ (to generate *Pdgfra*-CreER^{T2}/miR-34a$^{i\Delta PC/i\Delta PC}$) mice exposed either to normoxia or hyperoxia compared to tamoxifen-treated *Pdgfra*-CreER^{T2}/miR-34a$^{wt/wt}$ controls.

Parameter	21% O_2	85% O_2			
	Pdgfra-CreER^{T2}/miR-34a$^{wt/wt}$	*Pdgfra*-CreER^{T2}/miR-34a$^{wt/wt}$		*Pdgfra*-CreER^{T2}/miR-34a$^{i\Delta PC/i\Delta PC}$	
	mean ± SD	mean ± SD	*P* value *vs.* 34a$^{wt/wt}$/21% O_2	mean ± SD	*P* value *vs.* 34a$^{wt/wt}$/85% O_2
V (lung) [cm^3]	0.23 ± 0.01	0.20 ± 0.01	0.0272	0.23 ± 0.02	0.0498
CV [*V* (lung)]	0.07	0.07		0.09	
V_V (par/lung) [%]	90.75 ± 3.44	90.17 ± 2.34	0.941	91.18 ± 2.41	0.835
V_V (non-par/lung) [%]	9.25 ± 3.44	9.83 ± 2.34	0.941	8.82 ± 2.41	0.835
N (alv, lung) 10^6	4.46 ± 0.55	1.22 ± 0.13	< 0.0001	2.11 ± 0.12	0.0032
N_V (alv/par) 10^7 [cm^{-3}]	2.15 ± 0.31	0.70 ± 0.13	< 0.0001	1.03 ± 0.08	0.0492
CV [N (alv/lung)]	0.12	0.11		0.06	
S_V [cm^{-1}]	780.10 ± 91.01	512.50 ± 41.80	< 0.0001	561.80 ± 48.21	0.467
S (alv epi, lung) [cm^2]	162.70 ± 23.58	90.57 ± 6.25	< 0.0001	115.83 ± 16.26	0.085
CV [S (alv epi, lung)]	0.14	0.07		0.14	
V_V (alv air) [%]	66.12 ± 6.94	73.24 ± 2.81	0.166	70.18 ± 6.58	0.688
V (alv air, lung) [cm^3]	0.14 ± 0.02	0.13 ± 0.01	0.625	0.14 ± 0.01	0.266
CV [*V* (alv air, lung)]	0.14	0.05		0.09	
V (sep, lung) [cm^3]	0.07 ± 0.02	0.05 ± 0.01	0.063	0.06 ± 0.01	0.302
CV [*V* (sep, lung)]	0.23	0.17		0.27	
τ (sep) [µm]	8.66 ± 1.06	10.55 ± 1.84	0.315	10.68 ± 2.65	0.994
CV [τ (sep)]	0.12	0.17		0.25	
MLI [µm]	34.56 ± 7.17	57.39 ± 3.82	0.0001	50.25 ± 6.01	0.172
CV [MLI]	0.21	0.07		0.12	

Abbreviations: *alv*, alveoli; *alv air*, alveolar airspaces; *alv epi*, alveolar epithelium; *CV*, coefficient of variation; *MLI*, mean linear intercept; *N*, number; N_V, numerical density; *non-par*, non-parenchyma; *par*, parenchyma; *S*, surface area; S_V, surface density τ (sep), arithmetic mean septal thickness; *V*, volume; V_V, volume density. Values are presented as mean ± SD, *n* = 5 lungs for each group. A one-way ANOVA with Tukey's *post-hoc* analysis was used to determine *P* values.

Appendix Table S4. Stereological analysis of lungs from P14 wild-type mice treated with a target site blocker cocktail targeting the miR-34a/*Pdgfra* interaction exposed to hyperoxia, compared to scrambled target site blocker-treated controls.

Parameter	85% O_2		
	S	TSB1,2	
	mean ± SD	mean ± SD	*P* value *vs.* S/85% O_2
V (lung) [cm^3]	0.24 ± 0.01	0.22 ± 0.02	0.096
CV [*V* (lung)]	0.044	0.111	
V_V (par/lung) [%]	92.35 ± 4.62	90.66 ± 2.78	0.502
V_V (non-par/lung) [%]	7.65 ± 4.62	9.34 ± 2.78	0.502
N (alv, lung) 10^6	1.32 ± 0.17	1.77 ± 0.27	0.017
N_V (alv/par) 10^7 [cm^{-3}]	0.60 ± 0.07	0.90 ± 0.09	0.0001
CV [N (alv/lung)]	0.116	0.102	
Sf_V [cm^{-1}]	498.71 ± 49.86	536.89 ± 32.61	0.189
Sf (alv epi, lung) [cm^2]	110.37 ± 12.89	105.28 ± 14.41	0.572
CV [S (alv epi, lung)]	0.11	0.136	
V_V (alv air) [%]	65.31 ± 3.46	68.75 ± 1.63	0.079
V (alv air, lung) [cm^3]	0.14 ± 0.01	0.13 ± 0.01	0.167
CV [*V* (alv air, lung)]	0.04	0.098	
V (sep, lung) [cm^3]	0.07 ± 0.01	0.06 ± 0.01	0.012
CV [*V* (sep, lung)]	0.12	0.095	
τ (sep) [µm]	13.97 ± 1.41	11.69 ± 1.23	0.026
CV [τ (sep)]	0.100	0.105	
MLI [µm]	52.91 ± 7.04	51.32 ± 2.39	0.646
CV [MLI]	0.13	0.046	

Abbreviations: *alv*, alveoli; *alv air*, alveolar airspaces; *alv epi*, alveolar epithelium; *CV*, coefficient of variation; MLI, mean linear intercept; *N*, number; N_V, numerical density; *non-par*, non-parenchyma; *par*, parenchyma; S, antimiR-scrambled; *Sf*, surface area; Sf_V, surface density; TSB1,2, mixture of target site blockers 1 and 2; τ (sep), arithmetic mean septal thickness; *V*, volume; V_V, volume density. Values are presented as mean ± SD, *n* = 5 lungs for each group. An unpaired Student's *t*-test was used to determine *P* values.

Appendix Table S5. Stereological analysis of lungs from P14 wild-type mice treated with antimiR-34a exposed either to normoxia or hyperoxia, compared to scrambled antimiR-treated controls.

Parameter	21% O_2		85% O_2			
	S	A34a	S		A34a	
	mean ± SD	mean ± SD	mean ± SD	*P* value *vs.* S/21% O_2	mean ± SD	*P* value *vs.* S/85% O_2
V (lung) [cm^3]	0.23 ± 0.04	0.23 ± 0.04	0.24 ± 0.01	0.952	0.27 ± 0.02	0.448
CV [*V* (lung)]	0.19	0.19	0.044		0.079	
V_V (par/lung) [%]	89.54 ± 2.36	93.65 ± 3.91	89.30 ± 7.11	0.486	91.11 ± 3.46	0.932
V_V (non-par/lung) [%]	11.10 ± 2.36	6.39 ± 3.91	10.74 ± 7.11	0.486	1.60 ± 3.46	0.926
N (alv, lung) 10^6	4.57 ± 0.79	3.83± 0.54	1.22 ± 0.21	< 0.0001	2.03 ± 0.4	0.041
N_V (alv/par) 10^7 [cm^{-3}]	1.94 ± 0.39	1.51 ± 0.31	0.57 ± 0.03	0.640	0.87 ± 0.08	0.901
CV [N (alv/lung)]	0.173	0.14	0.172		0.203	
Sf_V [cm^{-1}]	940.40 ± 53.44	849.86 ± 72.18	450.66 ± 9.98	0.056	501.24 ± 48.1	0.426
Sf (alv epi, lung) [cm^2]	193.48 ± 42.09	179.51 ± 22.96	96.15 ± 4.84	0.805	123.24 ± 9.61	0.335
CV [S (alv epi, lung)]	0.22	0.13	0.050		0.078	
V_V (alv air) [%]	47.80 ± 4.51	52.89 ± 3.37	59.85 ± 8.93	0.501	74.93 ± 4.01	0.003
V (alv air, lung) [cm^3]	0.1 ± 0.02	0.11 ± 0.02	0.13 ± 0.01	0.751	0.184 ± 0.01	0.003
CV [*V* (alv air, lung)]	0.27	0.12	0.136		0.087	
V (sep, lung) [cm^3]	0.10 ± 0.01	0.10 ± 0.01	0.08 ± 0.02	0.951	0.06 ± 0.01	0.205
CV [*V* (sep, lung)]	0.17	0.19	0.263		0.190	
τ (sep) [µm]	11.10 ± 0.72	11.15 ± 1.3	17.81 ± 3.97	0.0001	10.15 ± 2.32	0.0007
CV [τ (sep)]	0.064	0.12	0.223		0.229	
MLI [µm]	20.44 ± 2.84	25.01 ± 2.35	53.15 ± 8.26	0.474	60.03 ± 3.74	0.159
CV [MLI]	0.13	0.09	0.15		0.06	

Abbreviations: A34a, antimiR-34a; *alv*, alveoli; *alv air*, alveolar airspaces; *alv epi*, alveolar epithelium; *CV*, coefficient of variation; *MLI*, mean linear intercept; *N*, number; N_V, numerical density; *non-par*, non-parenchyma; *par*, parenchyma; S, scrambled-antimiR; *Sf*, surface area; Sf_V, surface density; τ (sep), arithmetic mean septal thickness; *V*, volume; V_V, volume density. Values are presented as mean ± SD, n = 5 lungs for each group. A one-way ANOVA with Tukey's *post-hoc* analysis was used to determine *P* values.

Appendix Table S6. Summary of the gene expression microarray data examining changes in mRNA levels after antimiR-34a or scrambled antimiR treatment of mice with concomitant normoxia or hyperoxia exposure.

Time-point	Comparison	FC>|2.0| | *P*(corr)<0.05	
		Up	Down
P5	21% O_2/S *vs*. 21% O_2/A34a	0	0
	21% O_2/S *vs*. 85% O_2/S	622	612
	85% O_2/S *vs*. 85% O_2/A34a	0	0
P14	21% O_2/S *vs*. 21% O_2/A34a	0	0
	21% O_2/S *vs*. 85% O_2/S	2,972	3,093
	85% O_2/S *vs*. 85% O_2/A34a	0	0

Abbreviations: A34a, antimiR-34a; FC, fold-change; *P*, post-natal day; *P*(corr), corrected *P*-value; S, scrambled antimiR. n = 4 lungs per group. A Welch's approximate t-test was used to determine *P* values which were corrected using the algorithm of Benjamini and Hochberg, to generate *P*(corr).

Appendix Table S7. Real-time RT-PCR primers employed in this study for mRNA expression analyses.

Gene	Forward	Reverse
Acta2	5′-GCACCACTATGTACCCTGGCA-3′	5′- TTAGAAGCATTTGCGGTGGACAATG-3′
Ankrd37	5′-GGCCAGTGTTGCCTCTCTG-3′	5′-AGGTCAGCACCTGTTTGCAG-3′
Ccl1	5′-CTGGGATTCACCTCAAGAACATC-3′	5′-CAGGGTCAAGGCAAGCCTC-3′
Ccl5	5′-TCAAGCTCGCCATTCATGC-3′	5′-TTGCGGCTATGACTGAGGAAG-3′
Pdgfra	5′-AGAGTTACACGTTTGAGCTGTC-3′	5′-GTCCCTCCACGGTACTCCT-3′
Polr2a	5′-CTAAGGGGCAGCCAAAGAAAC-3′	5′-CCATTCAGCATACAACTCTAGGC-3′

Appendix Table S8. PCR primers employed for genotyping in this study.

Allele	Amplicon size	Forward	Reverse
Mir34a$^{tm1.2Aven}$	Mutant: 318 bp<br>WT: 198 bp	5′-GGTCACAAGACCCTCACCTG-3′	5′-TCACAGCAGACCCTTGATGT-3′
Mir34b/Mir34c$^{tm1.1Aven}$	WT: 377 bp	5′-AAATTCCTCCGACTGAGCCT-3′	5′-ATGACTTTACGGGGTTGACAG-3′
	Mutant: 249 bp	5′-TTGCGGGAAGAAGGACTC-3′	5′-ATGACTTTACGGGGTTGACAG-3′
Mir34a$^{tm1.1Lhe}$	WT: 471 bp	5′-ACTGCTGTACCCTGCTGCTT-3′	5′-GTACCCCGACATGCAAACTT-3′
	Mutant: 180 bp	5′-ACTGCTGTACCCTGCTGCTT-3′	5′-GCAGGACCACTGGATCATTT-3′
Tg(*Pdgfra*-cre/ERT2)1Wdr	Mutant: 500 bp	5′-GGTGTTATAACGAATCCCCAGAA-3′	5′-CAGGTCTCAGGAGCTATGTCCAATTTACTGACC-3′
B6.129S4-*Pdgfra*$^{tm11(EGFP)Sor}$/J	WT: 451 bp	5′-CCCTTGTGGTCATGCCAAAC-3′	5′-CCCTTGTGGTCATGCCAAAC-3′
	Mutant: 242 bp	5′-CCCTTGTGGTCATGCCAAAC-3′	5′-ACGAAGTTATTAGGTCCCTCGAC-3′

Abbreviations: WT, wild-type.

Appendix Table S9. Antibodies employed in this study.

Antigen	Host animal	Conjugated fluorophore	Supplier	Catalog Number	Application	Dilution
Aquaporin5	Rabbit	none	Abcam	Ab78486	IF	1:100
αSMA	Mouse	FITC	Sigma-Aldrich	F3777	FACS	1:100
βactin	Rabbit	none	Cell Signalling	4967	IB	1:4000
CD140a (PDGFRα)	Goat	None	R&D Systems	AF1062	IB	1:1000
CD140a (PDGFRα)	Rabbit	none	Cell Signalling	3174	IB	1:1000
CD140a (PDGFRα)	Rat	APC	Biolegend	135907	FACS	1:100
CD140a (PDGFRα)	Rat	PE	Biolegend	135905	FACS	1:100
CD31	Rat	none	Biolegend	102404	FACS	1:100
CD31	Rat	Pacific Blue	Biolegend	102422	FACS	1:100
CD31	Rat	PE	Biolegend	102407	FACS	1:100
CD326	Rat	none	Biolegend	118204	FACS	1:100
CD326	Rat	APC/Cy7	Biolegend	118217	FACS	1:50
CD326	Rat	FITC	Biolegend	118207	FACS	1:100
CD45	Rat	none	Biolegend	103104	FACS	1:100
CD45	Rat	APC/Cy7	Biolegend	103114	FACS	1:100
CD45	Rat	FITC	Biolegend	103108	FACS	1:100
CD71	Rat	PE	Biolegend	113808	FACS	1:100
c-Kit	Rabbit	none	Cell Signalling	3074	IB	1:1000
GAPDH	Rabbit	none	Cell Signalling	2118	IB	1:1000
Ki67	Mouse	PE	BD Pharmingen	556027	FACS	undiluted
Ki67	Rat	none	Thermo Scientific	14-5698-82	IF	1:100
Mouse IgG1κ	Mouse	FITC	Biolegend	400109	FACS	1:100
Mouse IgG1κ	Mouse	Pacific Blue	Biolegend	400131	FACS	1:100
Mouse IgG2aκ	Mouse	FITC	Biolegend	400207	FACS	1:100
ProSP-C	Rabbit	none	Abcam	Ab3786	IF	1:100
Rat IgG2aκ	Rat	none	Biolegend	400504	FACS	1:100
Rat IgG2aκ	Rat	none	Santa Cruz	Sc-2026	IF	1:100
Rat IgG2aκ	Rat	PE	Biolegend	400508	FACS	1:100
Rat IgG2aκ	Rat	PE/Cy7	Biolegend	400522	FACS	1:100
Rat IgG2aκ	Rat	APC	Biolegend	400511	FACS	1:100
Rat IgG2aκ	Rat	APC/Cy7	Biolegend	400523	FACS	1:50
Rat IgG2bκ	Rat	PE/Cy7	Biolegend	400617	FACS	1:100
Rabbit IgG isotype	Rabbit	none	Thermo Scientific	PA5-23090	IF	1:100
SIRT1	Rabbit	none	Cell Signalling	2493	IB	1:1000
Syrian Hamster IgG	Syrian hamster	PE/Cy7	eBioscience	25-4914-82	FACS	1:20
T1α	Syrian hamster	PE	Biolegend	127407	FACS	1:20

Abbreviations: APC, allophycocyanin; Cy7, cyanine 7; FITC, fluorescein; IF, immunofluorescence; PE, phycoerythrin; IB, immunoblot.

Appendix Figure S1

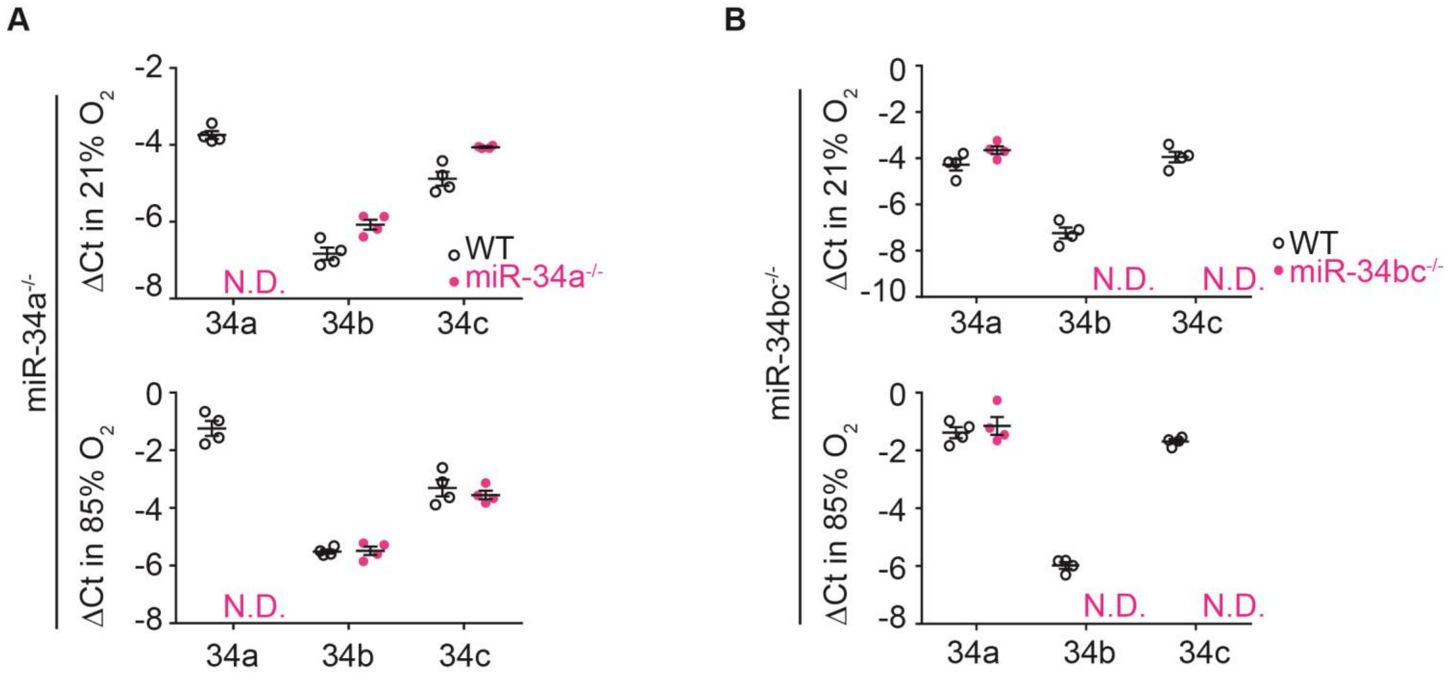

Appendix Figure S1. Loss of one member of the miR-34 family does not lead to compensatory upregulation of expression of other miR-34 family members. (A) Expression of miR-34a-5p (34a), miR-34b-5p (34b), and miR-34c-5p (34c) in wild type (WT) mouse lungs and miR-$34a^{-/-}$ mice after exposure to 21% O_2 (upper panel) or 85% O_2 (lower panel) from the day of birth up to and including the fifth day of life. (B) Expression of 34a, 34b, and 34c in WT mouse lungs and miR-$34bc^{-/-}$ mice after exposure to 21% O_2 (upper panel) or 85% O_2 (lower panel) from the day of birth up to and including the fifth day of life. Data represent mean ± SD ($n = 4$ mice, per group). Expression levels of each microRNA species within each WT/knockout pair were compared by unpaired Student's *t*-test. No significant difference ($P < 0.05$) in expression level comparisons was detected in any case; hence no *P* values are reported in the figure. No statistical comparison was possible when no amplicons were detected in one of the two experimental groups constituting the pair of data. N.D., no amplicon detected.

Appendix Figure S2

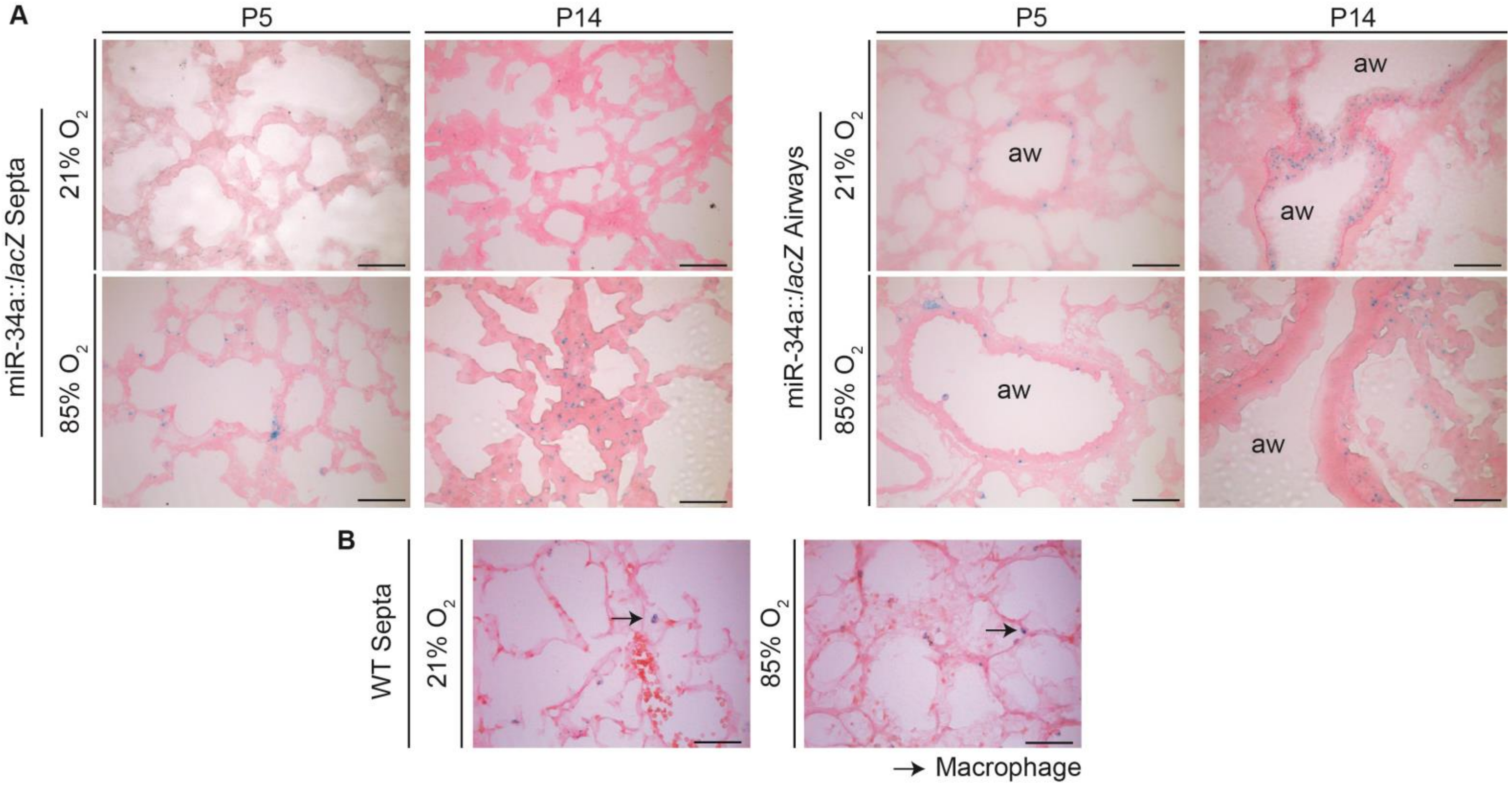

Appendix Figure S2. Expression of miR-34a was detected in the developing septa and airway epithelium in mouse pups. (A) Expression of miR-34a was localized using a miR-34a::*lacZ* reporter mouse strain, where β-galactosidase activity was revealed using 5-bromo-4-chloro-3-indolyl-β-D-galactopyranoside as substrate. The miR-34a expression is indicated by punctate blue foci in the developing septa (left panel) and in the developing airways (right panel). (B) It is noteworthy that macrophages (indicated by an arrow) stain positive for β-galactosidase activity even in wild-type (WT) mouse lungs. Data are representative of trends observed in two other independently-evaluated mouse lungs. Scale bar, 50 µm. aw, airway, P, post-natal day (as in P5 and P14).

Appendix Figure S3

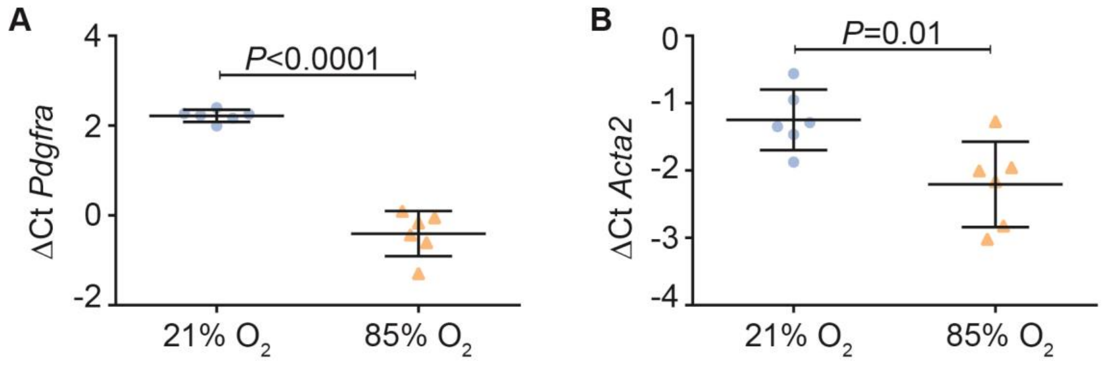

Appendix Figure S3. Exposure of the MLg mouse lung fibroblast cell line to 85% O_2 results in pronounced downregulation of expression of *Pdgfra* and *Acta2*. The MLg mouse lung fibroblast cell line was exposed to 85% O_2 in the incubator headspace for 48 h, after which mRNA levels for (A) *Pdgfra* and (B) *Acta2* were assessed by real-time RT-PCR. Data represent mean ± SD (n = 6 cell cultures for each group). P values were determined by unpaired Student's t-test.

Appendix Figure S4

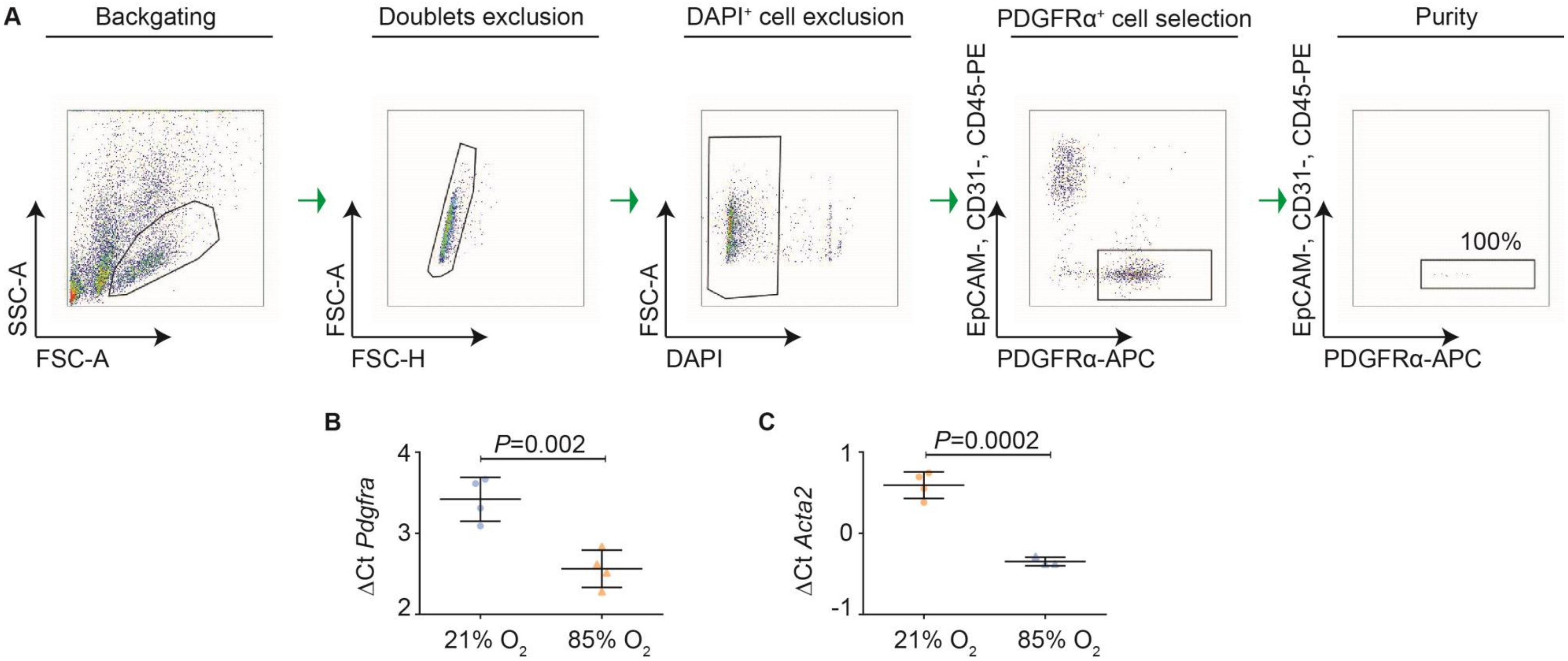

Appendix Figure S4. Exposure of mice to 85% O_2 downregulates *Pdgfra* and *Acta2* expression in PDGFRα$^+$ cells isolated from mouse lungs by flow cytometry. (A) Schematic illustration of PDGFRα$^+$ cell isolation from single-cell suspensions prepared from mouse lungs harvested from mice at post-natal day 5. The protocol illustrates backgating, doublet exclusion, fractionation of the cell population, and purity assessment. Note: an enrichment step was performed between panel 3 and panel 4, using magnetic bead-coupled anti-APC antibodies to pulldown the anti-PDGFRα-APC conjugate. The FACS-sorted PDGFRα$^+$ cells were assessed for the expression of (B) *Pdgfra* and (C) *Acta2* by real-time RT-PCR. Data represent mean ± SD (n = 4 animals for each group). P values were determined by unpaired Student's t-test. APC, allophycocyanin; DAPI, 4′,6-diamidino-2-phenylindole; FSC-A, forward scatter area; FSC-H, forward scatter height; PE, phycoerythrin; SSC-A, side scatter area.

Appendix Figure S5

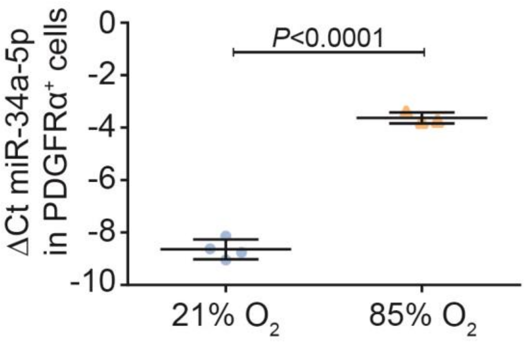

Appendix Figure S5. Repetition of the experiment presented in Fig. 3F. Quantitative RT-PCR detection of miR-34a-5p levels in PDGFRα$^+$ cells, sorted by FACS from the lungs of mouse pups (n = 4 animals for each group) at P5, maintained under 21% O_2 or 85% O_2 from birth. Data represent mean ± SD. P values were calculated by unpaired Student's t-test.

Appendix Figure S6

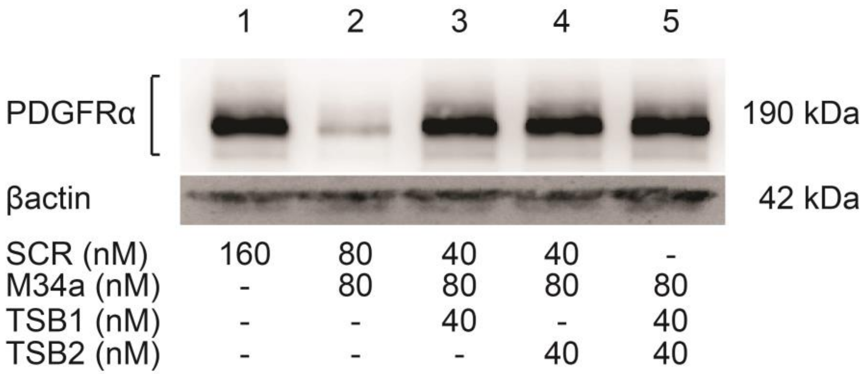

Appendix Figure S6. Validation of a target site blocker cocktail. Evaluation of the specificity of TSB1 and TSB2 used separately and together as a 1:1 cocktail in MLg cells, using scrambled miR (SCR) and miR-34a (MIM34a) mimics, and probing for PDGFRα. Protein loading equivalence was controlled by βactin levels. Note: PDGFRα and βactin were both probed on the same membrane. Data are representative of trends seen in two other experiments. **Source data exists for this figure.**

Appendix Figure S7

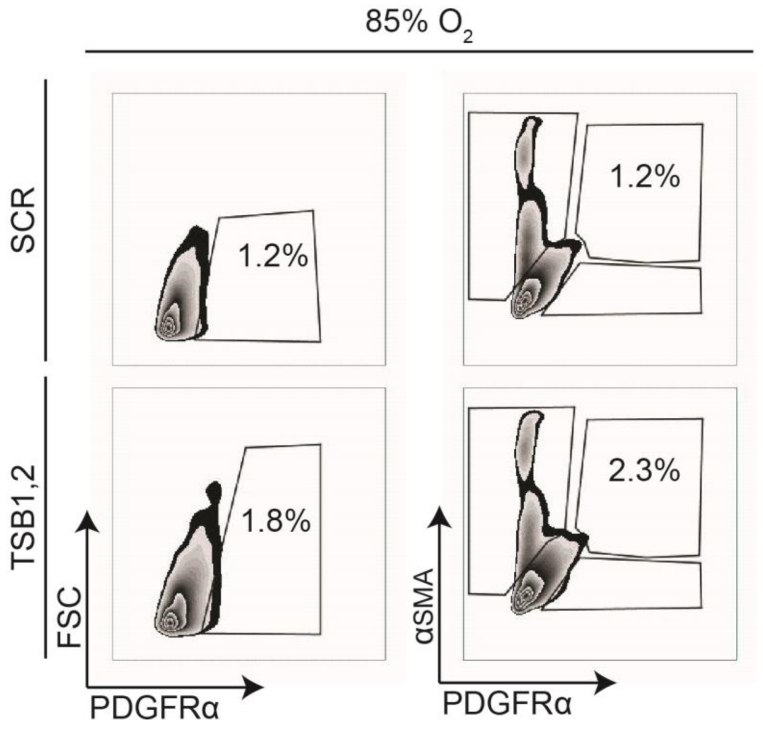

Appendix Figure S7. Gating strategy for the quantification of PDGFRα$^+$ and PDGFRα$^+$/αSMA$^+$ cells by flow cytometry in mouse lungs after target site blocker application. Illustration of the gating strategy to assess the impact of target site blocker (TSB)1 and TSB2 administration *vs.* scrambled TSB administration on the abundance of PDGFRα$^+$ and PDGFRα$^+$/αSMA$^+$ double-positive cells (myofibroblasts) in the lungs of P5 mice undergoing aberrant (85% O_2) alveolarization. FSC, forward scatter; PDGFRα, platelet-derived growth factor receptor α; SCR, scrambled target site blocker; TSB1,2, cocktail of two target site blockers designed to interrupt in interaction between miR-34a and *Pdgfra* mRNA.

Appendix Figure S8

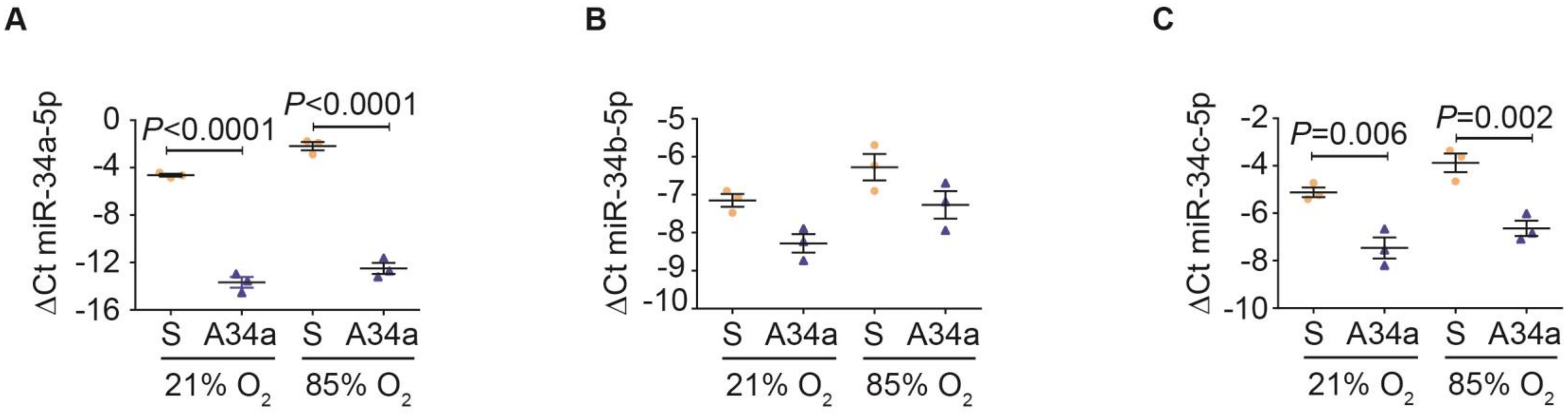

Appendix Figure S8. Administration of an antimiR directed against miR-34a predominantly impacts miR-34a expression in mice on day five of life. The impact of administration of scrambled antimiR (S) or an antimiR directed against miR-34a (A34a) on (A) miR-34a-5p, (B) miR-34b-5p, and (C) miR-34c-5p levels in the lungs of mice at post-natal day 5 (n = 3 animals for each group) undergoing normal (21% O_2) or aberrant (85% O_2) alveolarization. Data represent mean ± SD. P values were calculated by one-way ANOVA with Tukey's *post hoc* test.

Appendix Figure S9

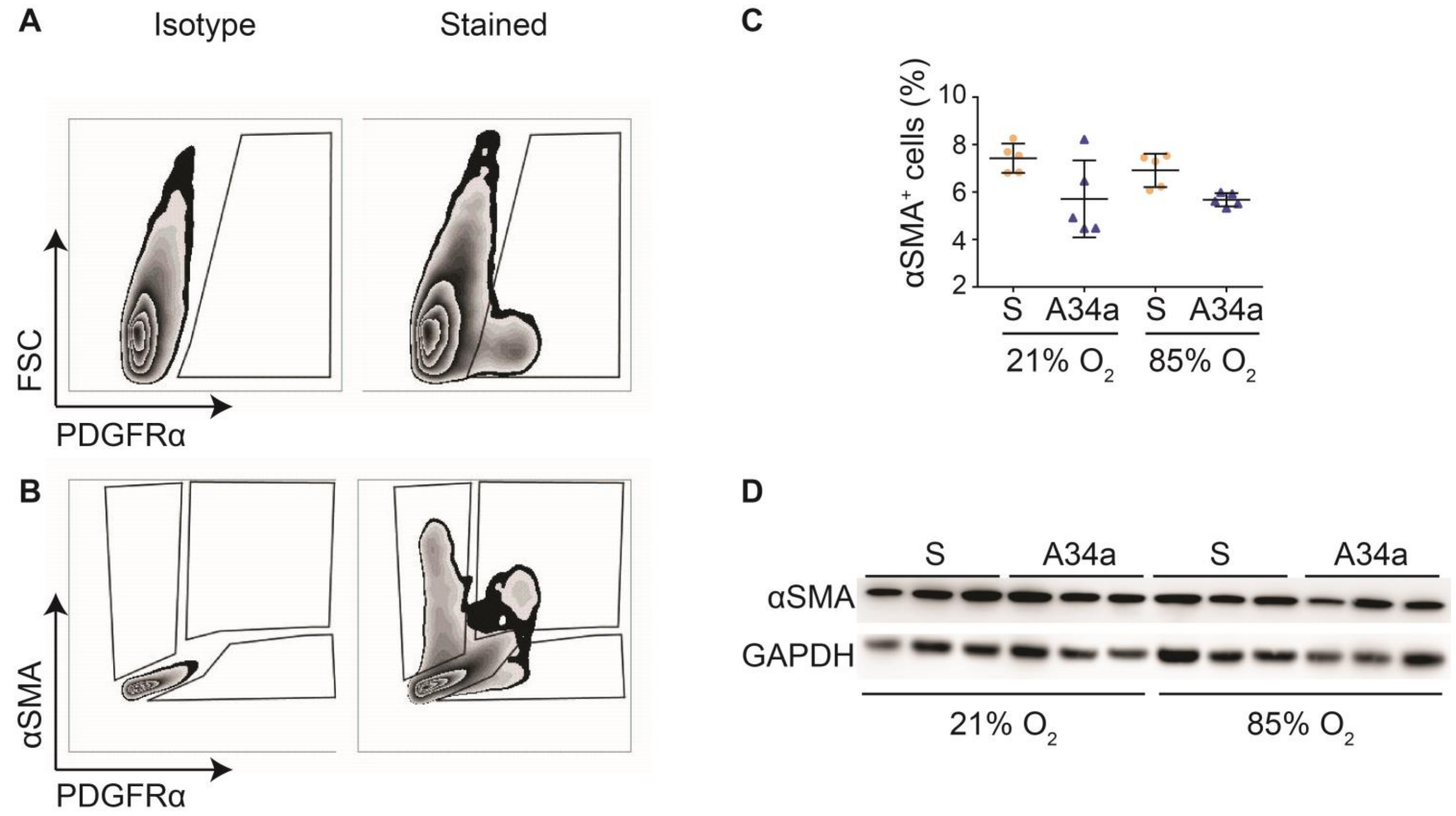

Appendix Figure S9. The abundance of α smooth muscle actin (SMA)-positive cells and total abundance of αSMA in mouse lung is not impacted by exposure of newborn mice to hyperoxia. Gating strategy for the detection of (A) platelet-derived growth factor receptor (PDGFR)α^+ and (B) PDGFRα^+/αSMA$^+$ cells isolated from the lungs of mice at post-natal day (P)5. (C) The impact of administration of scrambled antimiR (S) or an antimiR directed against miR-34a (A34a) on the abundance of αSMA$^+$ cells in the lungs of P5 mice undergoing normal (21% O_2) or aberrant (85% O_2) lung alveolarization. Data represent mean ± SD. (n = 5 animals/group; each data point represents an individual animal). No statistically significant ($P < 0.05$) differences in the abundance of αSMA$^+$ cells was detected by one-way ANOVA with Tukey's *post hoc* test, comparing all four experimental groups. (D) Protein expression levels of αSMA in the lung were detected by immunoblot in protein extracts from lungs of P5 mice under the same experimental conditions described in (C). Protein loading equivalence was controlled by GAPDH levels. **Source data exists for this figure.**

Appendix Figure S10

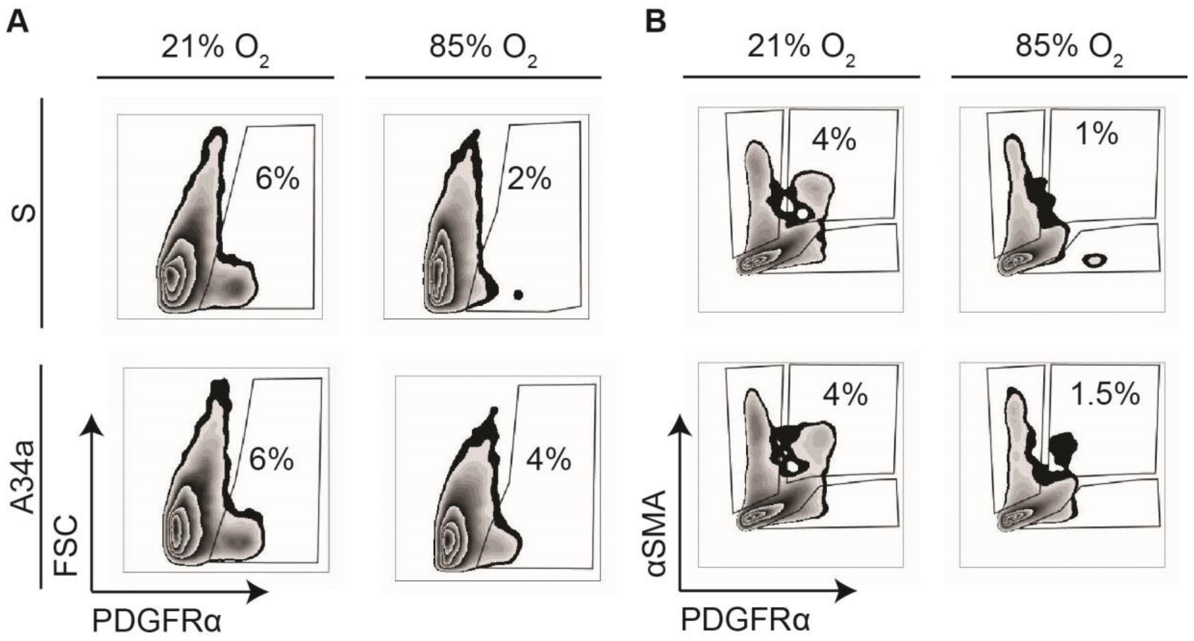

Appendix Figure S10. Gating strategy for the quantification of PDGFRα$^+$ and PDGFRα$^+$/αSMA$^+$ cells by flow cytometry in mouse lungs after antimiR-34a application. Illustration of the gating strategy to assess the impact of antimiR-34a (A34a) administration *vs.* scrambled antimiR (S) administration on the abundance of PDGFRα$^+$ and PDGFRα$^+$/αSMA$^+$ double-positive cells (myofibroblasts) in the lungs of P5 mice undergoing aberrant (85% O_2) alveolarization. A34a, antimiR-34a; FSC, forward scatter; PDGFRα, platelet-derived growth factor receptor α; S, scrambled antimiR.

Appendix Figure S11

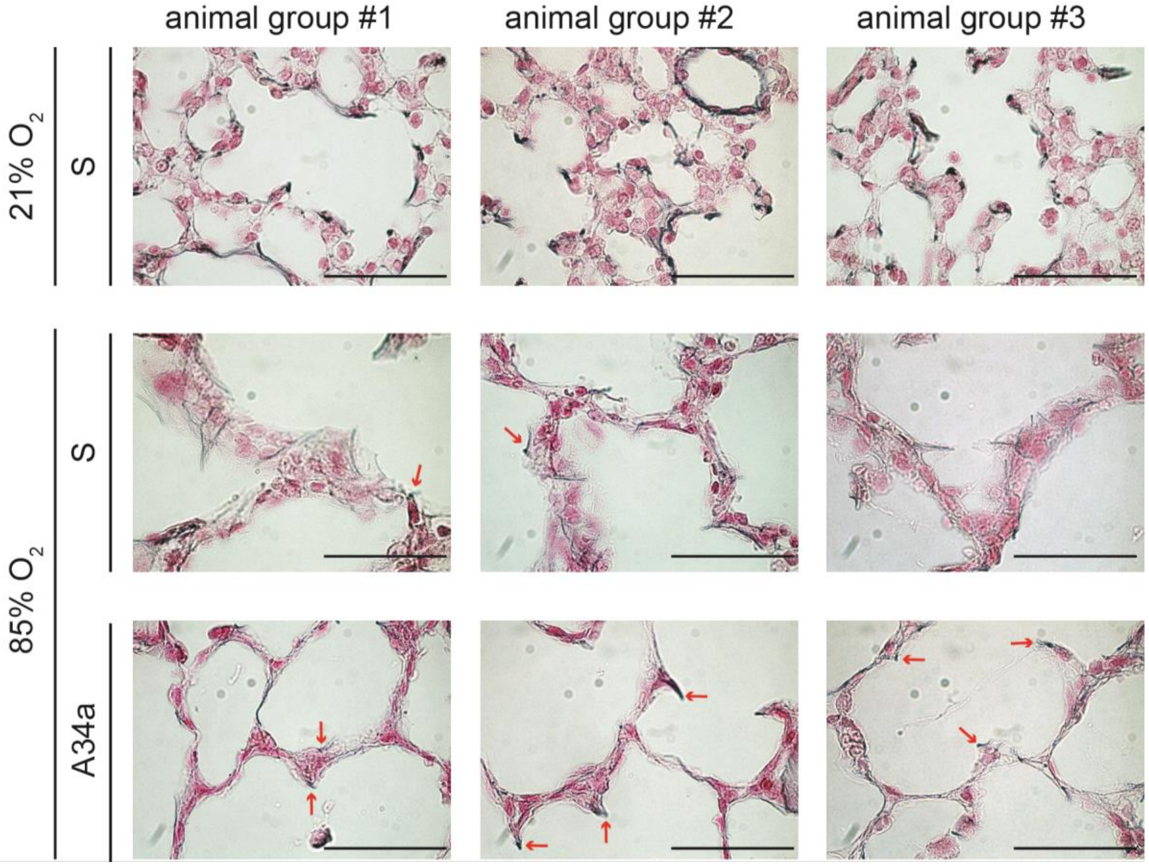

Appendix Figure S11. Administration of an antimiR directed against miR-34a impacts elastin deposition and remodeling during exposure of newborn mice to hyperoxia. Wild-type mice were treated with a scrambled (inert) antimiR sequence (S), or an antimiR directed against miR-34a (A34), on the first and third days of life, during continuous exposure to hyperoxia (85% O_2) for the first 14 days of life. For reference purposes, scrambled antimiR-treated mouse pups maintained under 21% O_2 for the first 14 days of life are also illustrated. Each of the three panel columns depict samples analyzed from different experimental animal groups (*n* = three independent experimental groups). Red arrows indicate selected examples of elastin foci. Scale bar, 50 µm.

Appendix Figure S12

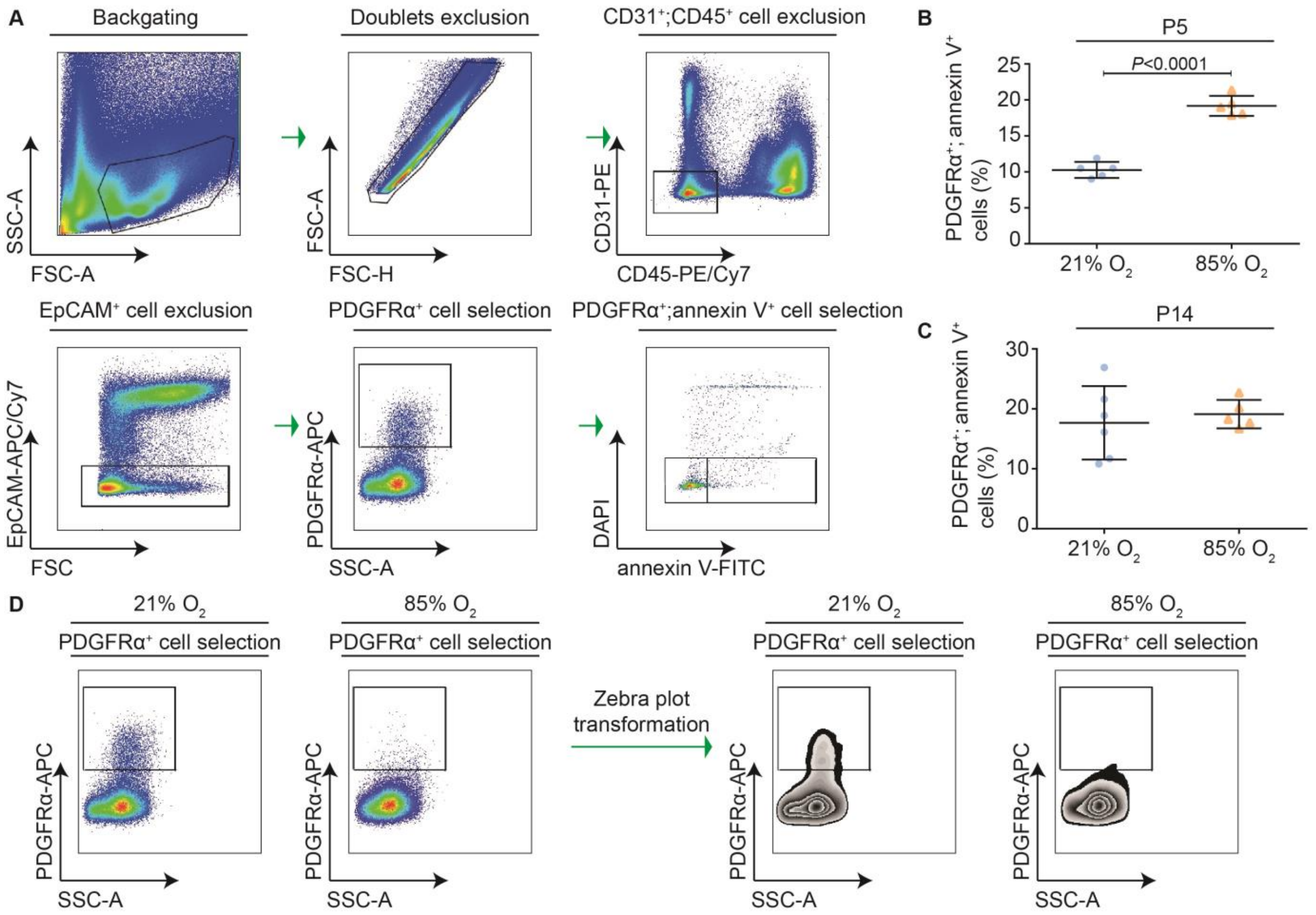

Appendix Figure S12. Determination of the apoptosis status of PDGFRα$^+$ cells in developing mouse lungs. (A) Protocol for the determination of apoptosis status of PDGFRα$^+$ cells in developing mouse lungs by flow cytometry. The apoptotic status of PDGFRα$^+$ cells in developing mouse lungs harvested at (B) P5 and (C) P4 was assessed by flow cytometry (P5, *n*=5 mice for each group; P14, *n*=6 mice for 21% O_2, and *n*=5 mice for 85% O_2). (D) Individual gating for PDGFRα$^+$ cells for mice exposed to 21% O_2 and 21% O_2 is indicated side-by-side (the 21% O_2 plot is duplicated from the middle row), and both plots are transformed into zebra plots to illustrate cell density. *P* values were determined by unpaired Student's *t*-test. APC, allophycocyanin; Cy7, cyanine 7; DAPI, 4′,6-diamidino-2-phenylindole; FITC, Fluorescein isothiocyanate; FSC-A, forward scatter area; FSC-H, forward scatter height; PE, phycoerythrin, SSC-A, side scatter area.

Appendix Figure S13

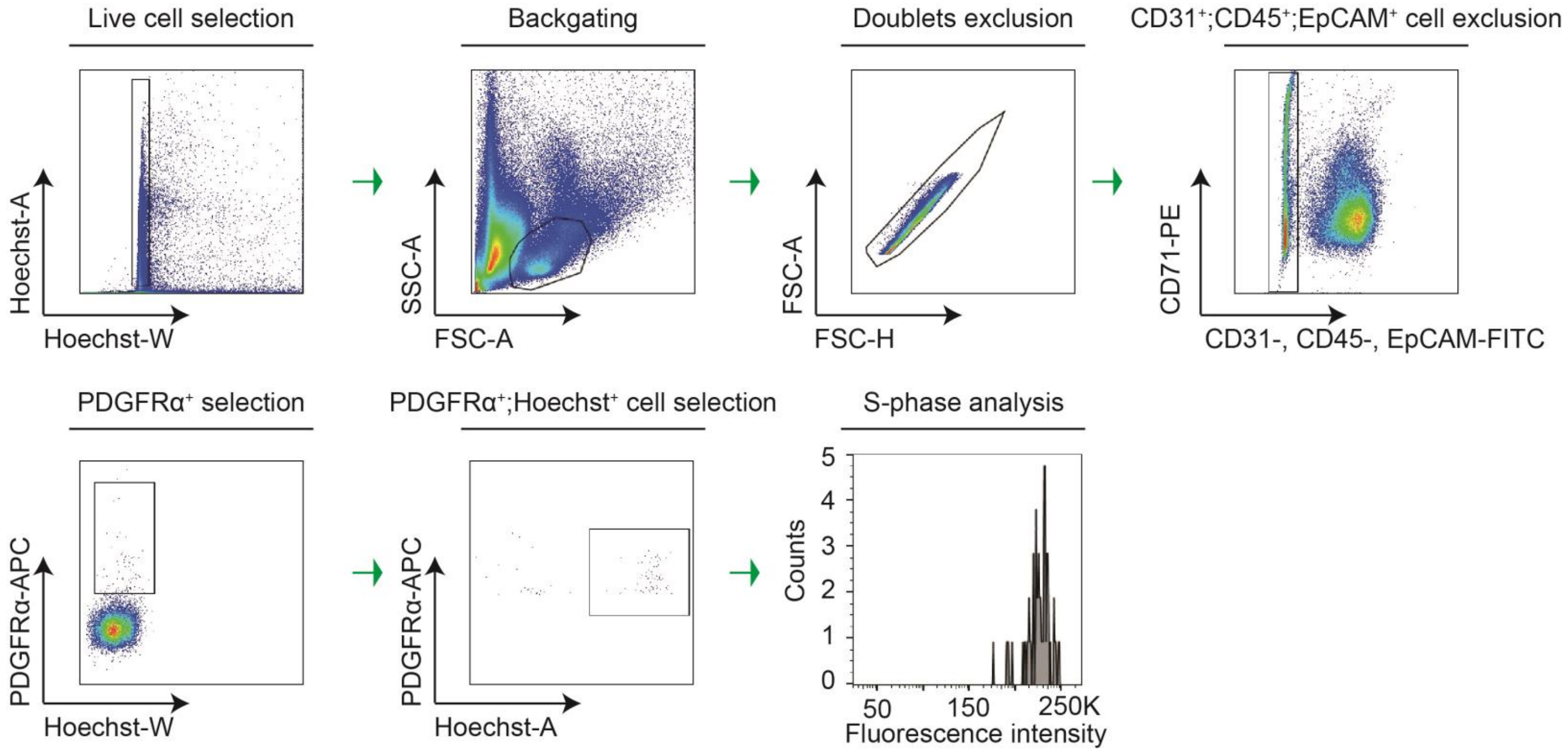

Appendix Figure S13. Attempt at the determination of the proliferation of status of PDGFRα$^+$ cells in developing mouse lungs by S-phase analysis. Protocol for the determination of proliferation status of PDGFRα$^+$ cells in developing mouse lungs by flow cytometry. APC, allophycocyanin; FSC, forward-scatter; FSC-A, forward-scatter area; PE, phycoerythrin; SSC-H, side-scatter height.

Appendix Figure S14

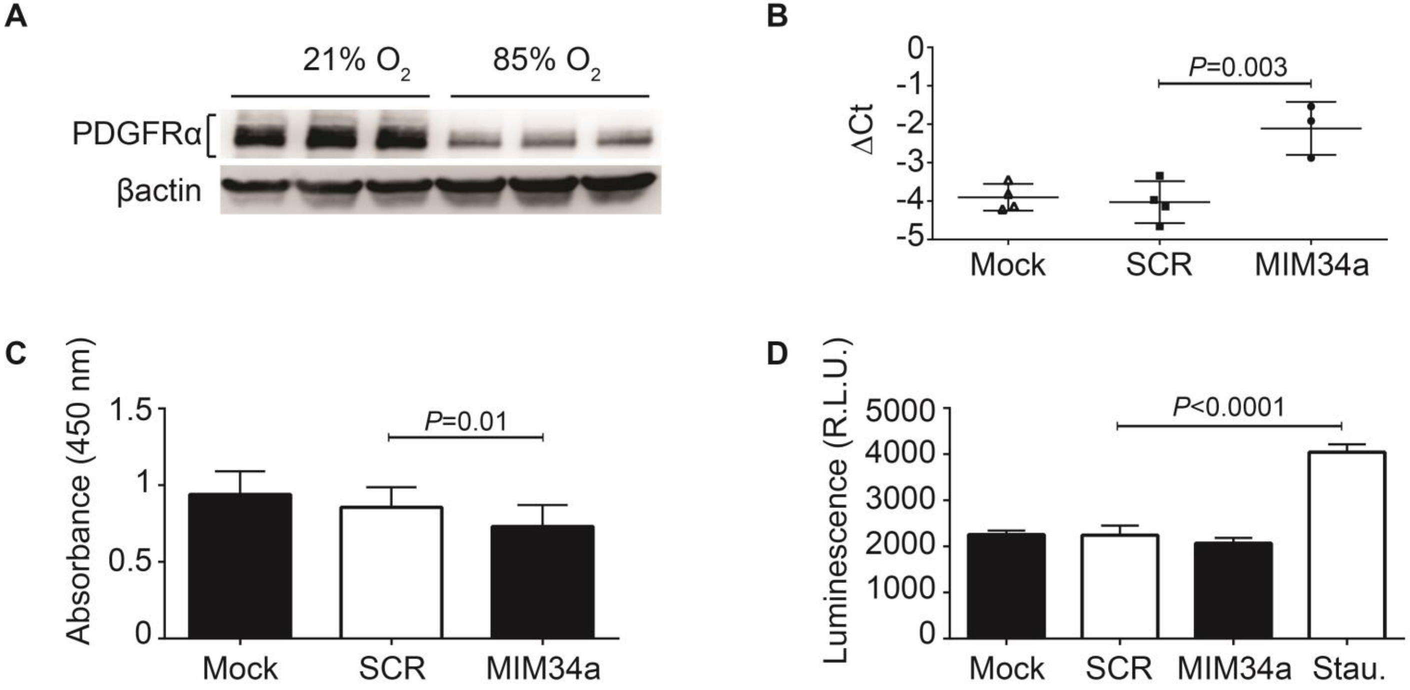

Appendix Figure S14. Determination of the impact of hyperoxia and miR-34a on apoptosis and proliferation of primary mouse lung fibroblasts. (A) The impact of hyperoxia exposure on PDGFRα levels in primary mouse lung fibroblasts was assessed my immunoblot, with βactin serving as a control for loading equivalence. (B) The impact of miR-34a-5p mimic (MIM34a) application to primary mouse lung fibroblasts on miR-34a-5p levels was assessed in mock-, scrambled (SCR) mimic- and MIM34a-treated cells by real-time RT-PCR analysis (*n*=4 for mock- and SCR-treatment, and *n*=3 for MIM34a treatment). Both (C) proliferation and (D) apoptosis were assessed in primary lung fibroblasts treated exactly as described in (B). Data reflect mean values ± SD (*n*=12 replicates per group, and data presented are representative of the trends observed in at least two independent experiments). R.L.U., relative luminescence units. *P* values were calculated by one-way ANOVA with Tukey's *post hoc* modification. **Source data exists for this figure.**

Appendix Figure S15

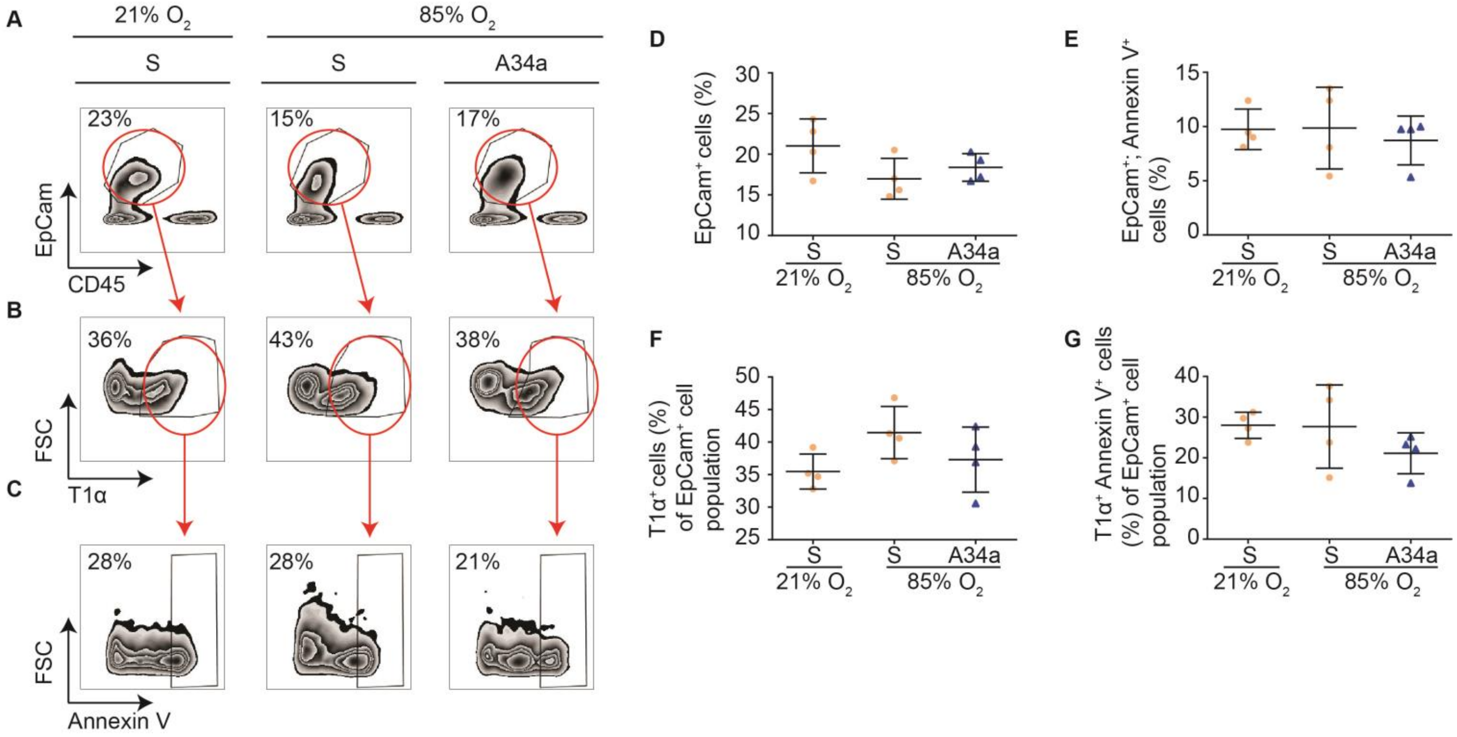

Appendix Figure S15. Neither hyperoxia exposure nor antimiR-34a treatment impacts total number of apoptotic status of alveolar type I cells. The impact of administration of scrambled antimiR (S) or an antimiR directed against miR-34a (A34a) on the abundance lung epithelial cells (marked by EpCam), type I alveolar epithelial cells (marked by T1α) and apoptotic cells (marked by annexin V) in lungs from P5 mice undergoing normal (21% O_2) or aberrant (85% O_2) alveolarization. The gating strategy for (A) EpCam$^+$ cells, (B) T1α$^+$ cells, and (C) annexin V$^+$ cells. Quantitative data are illustrated for (D) epithelial cells, (E) apoptotic epithelial cells, (F) type I alveolar epithelial cells, and (G) apoptotic type I alveolar epithelial cells. Data represent mean ± SD (n = 4 animals for each group). Cell percentages were compared by one-way ANOVA with Tukey's *post hoc* test. No significant difference ($P < 0.05$) in cell abundance comparisons was detected in any case; hence no P values are reported in the figure. FSC, forward scatter.

Appendix Figure S16

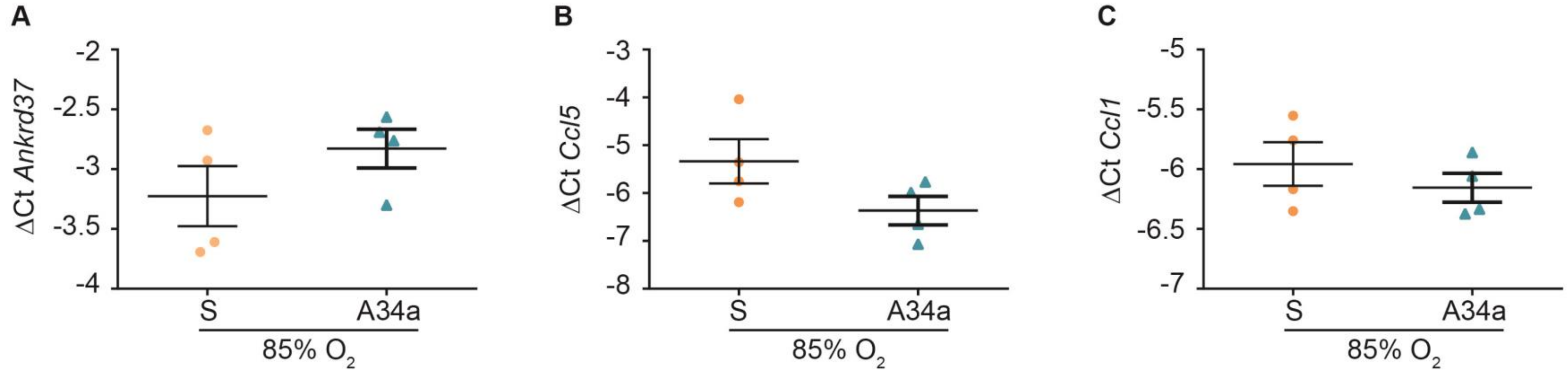

Appendix Figure S16. Administration of an antimiR directed against miR-34a does not have a detectable impact on mRNA expression in developing mouse lungs exposed to hyperoxia. A microarray analysis of the transcriptome of the lungs of mice treated with a scrambled antimiR (S) or an antimiR directed against miR-34a (A34a) that were undergoing normal (exposed to 85% O_2) and aberrant lung development (exposed to 85% O_2) was undertaken. Selected genes from this microarray are reported in Appendix Table S6 (full data set available at the GEO database under accession number GSE89730). Changes in the gene expression of candidate genes were validated by real-time RT-PCR: (**a**) *Ankrd37*, (**b**) *Ccl5*, and (**c**) *Ccl1*. Data represent mean ± SD. Expression levels of each candidate mRNA was compared by unpaired Student's *t*-test. No significant differences ($P < 0.05$) in expression level comparisons were detected in any case; hence no *P* values are reported in the figure.
