# Supplementary material for: Targeting miR‐34a/Pdgfra interactions partially corrects alveologenesis in experimental bronchopulmonary dysplasia
Source: EMBO Mol Med. 2019 Feb 15;11(3):e9448. doi: 10.15252/emmm.201809448 (PMC6404112; doi:10.15252/emmm.201809448)
Supplement: Supplementary file 2 — Source Data for Appendix [file EMMM-11-e9448-s004.zip › EMM-2018-09448-V3_SDataAppendixFigs/EMM-2018-09448-V3_SData_FigS6.pdf]

Source Data Appendix Figure S6

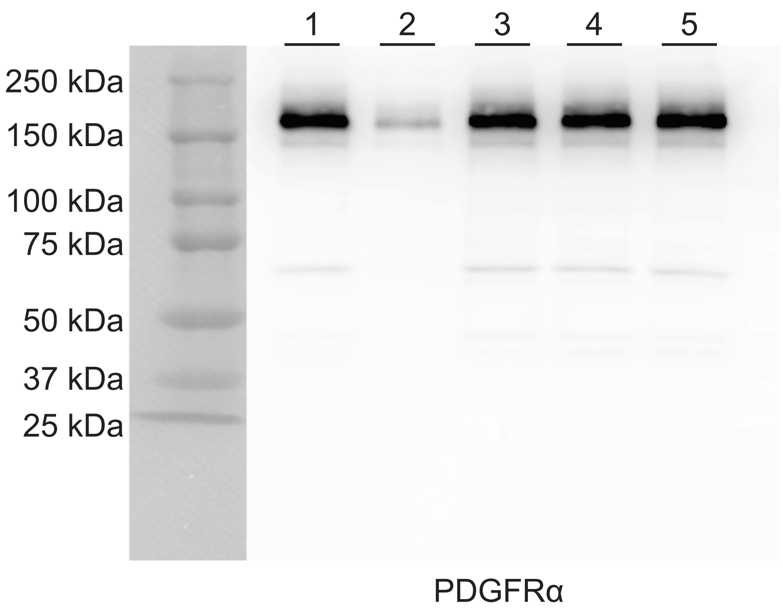

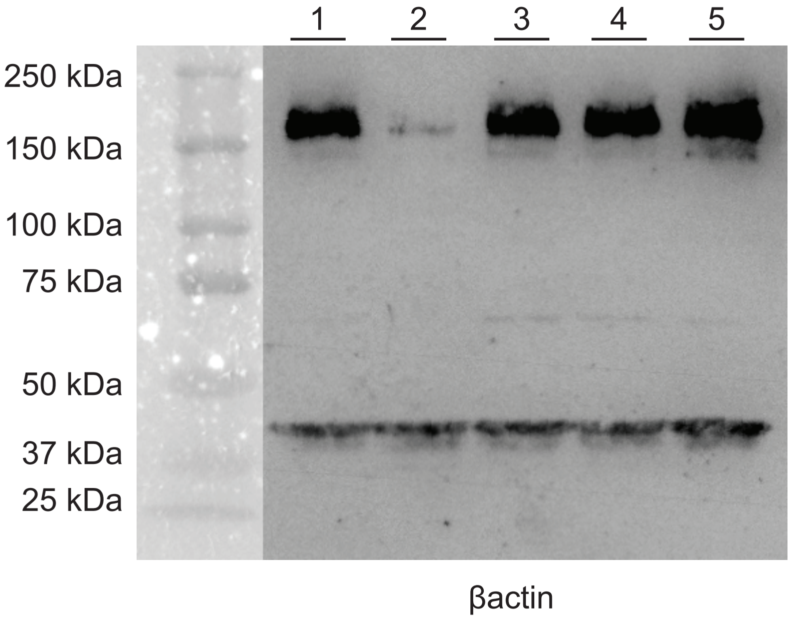

Procedure for alignment of protein ladder and immunoblot: The entire blot was digitally captured under white-light illumination (where the colored protein molecular mass marker ladder was visualized), and digitally captured again for chemiluminescent detection (for immunoblot bands). The blot contours were used to align the protein ladder alongside the immunoblot bands, as described graphically in the source data file for Figure 3.
